# Amputation of the Cervix Uteri with the Ecraseur

**Published:** 1857-08

**Authors:** 


*Amputation of the Cervix Uteri with the Ecraseur.*—Dr. McClintokc recently performed this operation with satisfactory results. But little blood was lost, and it is for this reason that the ecraseus was employed.—*New York Journal of Medicine.*

